# #Fail: the quality and accuracy of nutrition-related information by influential Australian Instagram accounts

**DOI:** 10.1186/s12966-024-01565-y

**Published:** 2024-02-14

**Authors:** Emily Denniss, Rebecca Lindberg, Laura E. Marchese, Sarah A. McNaughton

**Affiliations:** 1 Institute for Physical Activity and Nutrition, School of Exercise and Nutrition Sciences, Deakin University, 221 Burwood Hwy, Burwood, VIC 3125 Australia; 2 School of Human Movement and Nutrition Sciences, University of Queensland, St Lucia, QLD 4067 Australia

**Keywords:** Social media, Nutrition misinformation, Information quality, Nutrition communication, Health communication

## Abstract

**Background:**

Social media is a popular source of information about food and nutrition. There is a high degree of inaccurate and poor-quality nutrition-related information present online. The aim of this study was to evaluate the quality and accuracy of nutrition-related information posted by popular Australian Instagram accounts and examine trends in quality and accuracy based on author, topic, post engagement, account verification and number of followers.

**Methods:**

A sample of posts by Australian Instagram accounts with ≥ 100,000 followers who primarily posted about nutrition was collected between September 2020 and September 2021. Posts containing nutrition-related information were evaluated to determine the quality and accuracy of the information. Quality was assessed using the Principles for Health-Related Information on Social Media tool and accuracy was assessed against information contained in the Australian Dietary Guidelines, Practice-based Evidence in Nutrition database, Nutrient Reference Values and Metafact.

**Results:**

A total of 676 posts were evaluated for quality and 510 posts for accuracy, originating from 47 Instagram accounts. Overall, 34.8% of posts were classified as being of poor quality, 59.2% mediocre, 6.1% good and no posts were of excellent quality. A total of 44.7% of posts contained inaccuracies. Posts authored by nutritionists or dietitians were associated with higher quality scores (β, 17.8, CI 13.94–21.65; *P* < 0.001) and higher accuracy scores (OR 4.69, CI 1.81–12.14, *P* = 0.001) compared to brands and other accounts. Information about supplements was of lower accuracy (OR 0.23, CI 0.10–0.51, *P* < 0.001) compared to information about weight loss and other nutrition topics. Engagement tended to be higher for posts of lower quality (β -0.59, *P* = 0.012), as did engagement rate (β -0.57, *P* = 0.016). There was no relationship between followers or account verification and information quality or accuracy and no relationship between engagement and accuracy.

**Conclusions:**

Nutrition-related information published by influential Australian Instagram accounts is often inaccurate and of suboptimal quality. Information about supplements and posts by brand accounts is of the lowest quality and accuracy and information posted by nutritionists and dietitians is of a higher standard. Instagram users are at risk of being misinformed when engaging with Australian Instagram content for information about nutrition.

**Supplementary Information:**

The online version contains supplementary material available at 10.1186/s12966-024-01565-y.

## Introduction

Poor diet quality is the leading preventable risk factor contributing to the global burden of non-communicable disease [1]. Dietary behaviours are complex and are influenced by a range of factors, including hunger, taste preferences, food availability, price, societal norms, and policy context [2]. Nutrition information environments, which encompass the media and advertising, can also exert an influence on dietary behaviours [2]. Social media has recently become a prominent part of the modern media environment and is a popular vehicle for advertising, marketing and information sharing. Fifty-nine percent of the global population are active on social media, [3] and social media advertising revenue was projected to reach $173 billion USD in 2022 [4]. Food and nutrition are popular topics on social media platforms, [5, 6] and marketing of food and supplement products has become prolific [7, 8]. There is a growing body of evidence that indicates food and nutrition content and marketing on social media has the power to influence food choice [9–12].

As the Internet has become more accessible, individuals have increasingly utilised it to source information about nutrition. Consumers seek nutrition information for various reasons including health management, curiosity, and interest [13]. Nutrition information is content that provides the general public with guidance on sourcing, storing, preparing and consuming food to support good health, and includes recipes, product details, healthy-eating advice and nutritional requirements (defined in full in Table 1). Increases in online nutrition information seeking behaviour have been observed in America, [14] Canada, [15] France, [16] and Norway, [17] and the Internet is the primary source of nutrition information for Australians [18, 19]. Social media has become a ubiquitous part of the Internet and consumers search for and follow food and nutrition-related content on social media [6, 20–22]. A survey of American Instagram users found that 87% of female users followed nutrition-related content on the platform [6]. Consumers not only seek information about food and nutrition on social media they are also passively exposed to it in their social media feeds without intentionally searching for it [23, 24]. Due to the influence of social media algorithms and paid sponsorships, social media users are also presented with content in their feed from entities that they do not follow. Furthermore, content can be published by anyone, regardless of their qualifications, level of expertise in the topic or conflicting interests.

**Table 1:** Key definitions

Accuracy: The factual correctness of information when compared to authoritative sources of information such as systematic reviews, meta analyses or national dietary guidelines [25].
Quality: The reliability of information as assessed by quality criteria [26]. Quality criteria typically include components such as readability, inclusion of references, transparency about financial interests and disclosure of the author’s identity and qualifications
^a^Nutrition-related information: information regarding healthy eating, dietary patterns, nutrients, nutritional requirements, nutritional composition of foods, nutritional supplements, health outcomes associated with foods and dietary patterns, food safety, food ethics, cooking and recipes intended for the general public [25].

^a^From Denniss et al. [25]

Accurate and high-quality information is essential for effective health communication and promotion. In health communication literature, accuracy refers to health information’s factual correctness, and quality refers to information’s reliability when assessed using defined quality criteria (see Table 1) [25, 26]. Quality and accuracy are two important but distinct components of information’s overall reliability. It is possible for information to be accurate but of low quality and vice versa. Previous studies have assessed the accuracy of online nutrition information against authoritative sources such as dietary guidelines, authoritative reports and peer reviewed literature [25]. Numerous quality assessment tools have been used to evaluate the quality of online nutrition information, such as the DISCERN Instrument, [27]. Journal of the American Medical Association Benchmarks, [28] and Health on the Net Code Principles [7, 29]. These tools share common quality criteria, such as, declaring financial interests, citing sources, authorship by an individual with relevant health-related qualifications, and disclosure of the author’s qualifications. Principles for Health-related Information on Social Media (PRHISM), is a recently developed quality assessment tool, which includes these established quality principles and additional principles that are relevant to social media, including accessibility and readability [30]. There is a broad consensus in the literature that health information of suboptimal quality and accuracy is extremely prevalent on social media [26, 31]. Concerningly, misinformation is often more popular than truthful information, receives higher user engagement and spreads more quickly than the truth due to its novelty [31, 32]. Furthermore, the narrative of health misinformation often includes and promotes mistrust in authoritative institutions and experts [31]. Social media-based misinformation can have consequences for public health, for example, the online anti-vaccination movement is believed to have contributed to a reduction in vaccination rates and the reemergence of previously eradicated communicable diseases [31, 33].

Public health nutrition experts and organisations have raised concerns about the potential for nutrition-related misinformation to cause serious harm and undermine credible nutrition communication. Exposure to nutrition information that lacks context or contradicts previous messaging can lead to confusion and backlash, which has been evidenced to reduce consumers’ willingness to engage in healthful behaviours and accept advice by authoritative nutrition experts [34, 35]. If dietary behaviours are based on misinformation that contradicts evidence-based dietary guidelines, it may put individuals at greater risk of developing non-communicable diseases that are associated with unhealthy dietary patterns [1]. A recent systematic review of studies evaluating the quality and accuracy of nutrition-related information published on websites and social media found that generally information was of suboptimal quality and accuracy [25]. A small number of studies in the review investigated certain social media (e.g., blogs, YouTube, Facebook, Twitter and WhatsApp), meaning other platforms, such as Instagram have had limited attention [25]. A further limitation was that quality assessment tools designed for different settings, for example, websites, were used to evaluate the social media content [25]. Furthermore, it was also rare for studies to involve multiple researchers when screening posts to evaluate, which is a potential source of bias [25]. One recent study that was not captured within the date-range of the systematic review examined the quality of nutrition-related information on Instagram finding that quality was extremely low [36]. However, this study measured quality as a single criterion, rather than using an extensive quality assessment method, and thus may not have comprehensively measured the quality of information.

To the knowledge of the authors, thus far no studies have assessed the accuracy of nutrition information on Instagram, and none have used social media specific tools to evaluate the quality of nutrition-related Instagram content. Instagram was the third most popular social media platform in Australia, and the fifth most visited website in the world in 2021, [3, 37] and nutrition is one of the most frequently discussed health topics on Instagram [5]. Therefore, the aim of this study was to evaluate the quality and accuracy of nutrition information posted by popular Australian Instagram accounts using the PRHISM tool to assess quality. A secondary aim was to examine trends in information quality and accuracy by author, topic, post engagement, account verification and number of followers.

## Methods

### Study design and data collection

The present study involved a cross-sectional evaluation of the quality and accuracy of nutrition-related information published on Instagram by Australian influencer or brand accounts. The Strengthening the Reporting of Observational Studies in Epidemiology (STROBE) guidelines were followed and the checklist is available in Supplementary Table 1 [38]. A subsample of Instagram posts collected for a wider project formed the dataset for this study and data collection has been described in detail elsewhere [7]. Briefly, a list of the top 1,000 Australian health Instagram accounts as of April 2021 was screened to determine eligibility [39]. Australian accounts with over 100,000 followers, a minimum of 100 posts, their most recent post published within two weeks, and a minimum of 25% of their content relating to nutrition were included. The figure of 100,000 followers was chosen because accounts with over 100,000 followers are considered to be macro influencers or above (over one million followers is considered to be a mega influencer) [40]. Social media users tend to trust the influencers and brands that they follow, [41] and the purpose of this study was to capture information posted by popular Australian Instagram accounts, therefore, only accounts with greater than 100,000 followers were considered eligible for inclusion. Accounts appearing under the “suggested” tab on the Instagram page of included accounts were also screened. Screening was done between May and July 2021 using an Instagram account made specifically for the project and using Google Chrome’s incognito mode to minimise the impact of the algorithm on the suggested accounts. Two researchers (ED and JV, named in acknowledgements) independently screened all accounts and disagreements were discussed until agreement was reached.

All posts by eligible accounts from a twelve-month period (September 2020 – September 2021) were downloaded through a paid subscription to Keyhole, an online social media analytics tool. The data extracted through Keyhole included each post’s text-based caption, engagement (sum of likes and comments), the date of upload and each account’s bio, number of followers and if it was verified with a blue tick. Each post was manually screened for relevance. Video content including Reels and posts that did not refer to one or more component of nutrition-related information, as defined in Table 1, were excluded. Ten percent of posts were screened independently by the lead author (ED) and a research assistant (JV) for relevance to nutrition information, with 94% agreement. The screening resulted in a sample of 10,964 Instagram posts containing nutrition-related information, which have been characterised in a previous content analysis study [7].

For this study, a random stratified (by Instagram account) subsample of the 10,964 posts were selected and screened further for eligibility. A total of 2,025 posts were randomly selected to be screened (up to 35 posts from each of the included Instagram accounts). The random subsample for screening was selected using the sample function in Stata in March 2022. Posts that promoted a product but did not include any information about the health benefits of the product, for example, promotion of a supplement without mention of its benefits, and posts that only included recipes or meal ideas and no additional information were excluded because these posts did not provide nutrition guidance that could be evaluated for accuracy or quality. All other posts were deemed eligible. Screening was done independently by two researchers (all posts screened by ED and with secondary screening by SM, LM or research assistant JL). Disagreements were discussed by the two researchers responsible for screening the relevant post until consensus was reached.

### Quality and accuracy evaluations

The quality of information was evaluated using the PRHISM tool [30]. PRHISM was developed as a tool to evaluate the quality of social media-based information about any health-related topic and considers the unique characteristics of social media content, for example, covert advertising and brevity of information. To the knowledge of the authors, PRHISM is the only quality assessment tool that has been specifically developed for health information delivered via social media. Other tools for evaluating the quality of health-related information exist. However, these tools were developed for different settings, such as websites or patient information pamphlets and their use to evaluate health-related social media content has been scrutinised in the literature [25, 42, 43]. PRHISM was developed in a Delphi study, which involved a panel of 18 expert participants [30]. The participants were experts in health communication or a health-related field, used social media to communicate about health and worked in academia, public health, communications and/or as health professionals. Three Delphi surveys were undertaken to determine group consensus about the principles to include in PRHISM. Principles were based on quality criteria from previous tools and adapted to suit social media or were new principles suggested by the expert panel. After the third Delphi survey, a draft of the PRHISM scoring tool and guide for use was circulated to participants for feedback about face validity and participants agreed with the final wording of the principles and guide [30].

PRHISM consists of thirteen principles that are scored from zero to four [30]. A number of principles may not be relevant to evaluate depending on the nature of the social media post being evaluated, for example, principle 8 relates to privacy and is only relevant to evaluate if the post discusses a client or patient. The overall PRHISM score is weighted proportionately as a percentage of the total available score, whereby principles that are deemed not relevant do not contribute to the total available score. The overall PRHISM score ranges from 0 – 100 where a higher score indicates higher quality. A score of zero to 25 is considered poor, 26– 50 mediocre, 51 – 75 good and 76 – 100 excellent quality [30]. All images and information contained within the post, information in the account holder’s bio and information contained in links provided within the post or bio were considered in quality evaluations, as outlined in the PRHISM protocol [30]. A summary of the PRHISM assessment principles is included in Table 2.

**Table 2:** Summary of quality principles assessed by the PRHISM tool and accuracy score categories

^a^PRHISM tool principles for quality assessments	
**Principle**	**Description**
1. Authorship	Clear information about the author’s credentials, qualifications and affiliations should be provided
2. Authoritative	The information should be authored by qualified professionals and within the author’s scope of practice
3. Action-oriented	Information should be clear and succinct and provide sufficient context to support consumer decision making
4. Financial disclosure	Clear and prominent disclosures of relevant sponsorship, advertising or financial support should be disclosed. Paid sponsorships should be made in a way that complies with the social media platform’s guidelines
5. Attribution	Information should include citations and hyperlinks to the original source of information and include the year the information was published
6. Balance and justifiability	Information should be balanced, unbiased and supported by high quality and appropriate evidence
7. Risks and benefits	Information should clearly outline all relevant health risks and benefits
8. Privacy	If information about a patient or client is shared, it should be shared with permission and not include any identifying information
9. Complementary information	Information should be complimentary and not designed to replace the relationship between individuals and health professionals. Information should include statements encouraging individuals to discuss choices with a relevant health professional
10. Referrals and support	Readers should be referred to additional sources of information and support
11. Readability and comprehensibility	Information should be written at or below a fifth grade reading level and avoid the use of jargon and technical language
12. Accessibility	Information should be accessible to vision and hearing-impaired individuals. Videos should contain closed captions and images should include descriptive alternative text. Information should be accessible with screen readers
13. Images	If information contains images they should be visually appealing and appropriately reflect the information provided within the post, rather than contradicting it
**Accuracy score categories**	
**Category**	**Description**
Completely accurate	All assessable statements within the post that relate to nutrition are completely accurate
Mostly accurate, some inaccurate	The majority of statements within the social media post that relate to nutrition are completely accurate. A smaller proportion of statements within the post are inaccurate or contain inaccuracies
Mostly inaccurate, some accurate	The majority of statements within the social media post that relate to nutrition are completely inaccurate or contain inaccuracies. A smaller proportion of statements within the post are accurate
Completely inaccurate	All assessable statements within the post that relate to nutrition are completely inaccurate
Not assessable	All information within the post that relates to nutrition is not contained in the reference documents or databases, is a testimonial or is information discussing someone’s own personal behaviour, rather than stating a fact

*Abbreviations*: *PRHISM*: Principles for Health-related Information on Social Media

^a^From: Denniss et al. [30].

To determine accuracy, the information contained in the Instagram posts was evaluated against information contained in the Australian Dietary Guidelines (ADG) [44] Nutrient Reference Values (NRVs), Practice-based Evidence in Nutrition Global Resource for Nutrition Practice (PEN), [45] and/or Metafact fact-checking platform. [46] Metafact is a website that enables the public to ask questions, which are answered by verified PhDs, researchers and medical specialists.[46] Multiple experts can answer the questions and if ≥ 70% of verified experts agree on an answer, it is deemed that consensus has been achieved [46]. Only questions with consensus were used for accuracy evaluations. A large number of posts within the sample contained claims about the benefits of collagen supplementation for skin health, however, relevant information was not contained within the chosen authoritative resources. Therefore, a recent systematic review about collagen supplementation was also used to determine the accuracy of claims about collagen and skin health [47]. An accuracy coding framework was developed and was informed by a systematic review of the quality and accuracy of online nutrition information [25]. Posts were coded as containing information that is completely inaccurate (0), mostly inaccurate with some accuracies (1), mostly accurate with some inaccuracies (2), completely accurate (3) or not assessable (4) (see Table 2 for descriptions of accuracy categories). All the nutrition information within a post was considered when evaluating accuracy. If posts contained information about nutrition and other topics, such as sleep or exercise, only the information about nutrition was evaluated. Some posts contained claims about the health benefits of specific products, such as supplements, but did not contain sufficient information about the nutrients or ingredients contained in the product for an assessment to be made. In these instances, additional information about the product was sourced from its website in order to evaluate the accuracy of the claims.

Assessments were conducted and recorded in a purpose-designed REDCap database. A set of coding rules was developed to ensure that information that appeared multiple times in the sample of posts was coded consistently for accuracy. Before completing the quality and accuracy evaluations, a random 10% if the sample was evaluated independently by two authors (ED and LM) for inter-rater reliability, achieving 79% agreement for quality category and 85% for accuracy score. Disagreements regarding quality or accuracy evaluations were discussed by ED and LM until consensus was reached. Common reasons for disagreement were discussed and relevant updates to the study protocol were made before the remaining evaluations were completed to improve rigor. ED conducted the quality and accuracy assessments for the remainder of the sample to further improve consistency. Posts containing information that was difficult to evaluate were discussed before reaching a decision. Instagram accounts and the topic of Instagram posts were inductively categorised. The categories for Instagram accounts were developed by the two researchers (ED and JV) who undertook the screening of Instagram accounts, after screening had concluded and both researchers were immersed in the data. Each included account was independently categorised, and disagreements were discussed by ED and JV until agreement was reached. The categories for Instagram accounts were used in the preceding study for which the data was originally collected [7]. Categories for post topic were developed during the reliability assessments based on the most frequent nutrition-related topics mentioned in the posts and all posts were categorised by the first author. Descriptions of account and topic categories are summarised in Supplementary Table 2.

### Statistical analysis

Data was exported from REDCap and statistical analyses were performed in Stata/SE v17.0 (StataCorp, College Station, TX). Descriptive statistics were run. Multilevel mixed-effects models were used to estimate quality scores for account categories, post topics, account verification, accuracy score, follower count, engagement, and engagement rate. Marginal means of quality scores for account categories and post topics were calculated. Pairwise comparisons were run to determine mean differences in quality scores across account categories, post topics and accuracy categories. Mixed-effects ordered logistic regression models were used to estimate accuracy score outcomes for account categories, post topics, account verification, follower count, engagement and engagement rate. Pairwise comparisons were run to determine mean differences in quality scores across account categories and post topics. Mixed-effects models were chosen to account for the structure of the data, with the possibility of repeat measures (i.e., multiple posts) for each Instagram account, with posts being the unit of analysis and the models including random intercepts for Instagram accounts. Quality and accuracy scores were treated as the dependent variable. Engagement and engagement rate had positively skewed distributions and were log transformed. One account had an outlying number of followers (> 2,000,000) and was thus removed from analyses involving follower counts. After removing the outlying account, follower counts remained skewed and were also log transformed to achieve normal distribution. Analysis was run with and without the outlying account showing little difference to the result (data not shown). Statistical significance was set at *P* < 0.05.

### Ethics

The Instagram posts included in this study were publicly available. Due to the public nature of the data, this study was exempt from formal review by an ethics committee. To ensure that this study upholds ethical research standards, no identifying information about the accountholders has been published.

## Results

**Fig. 1:**
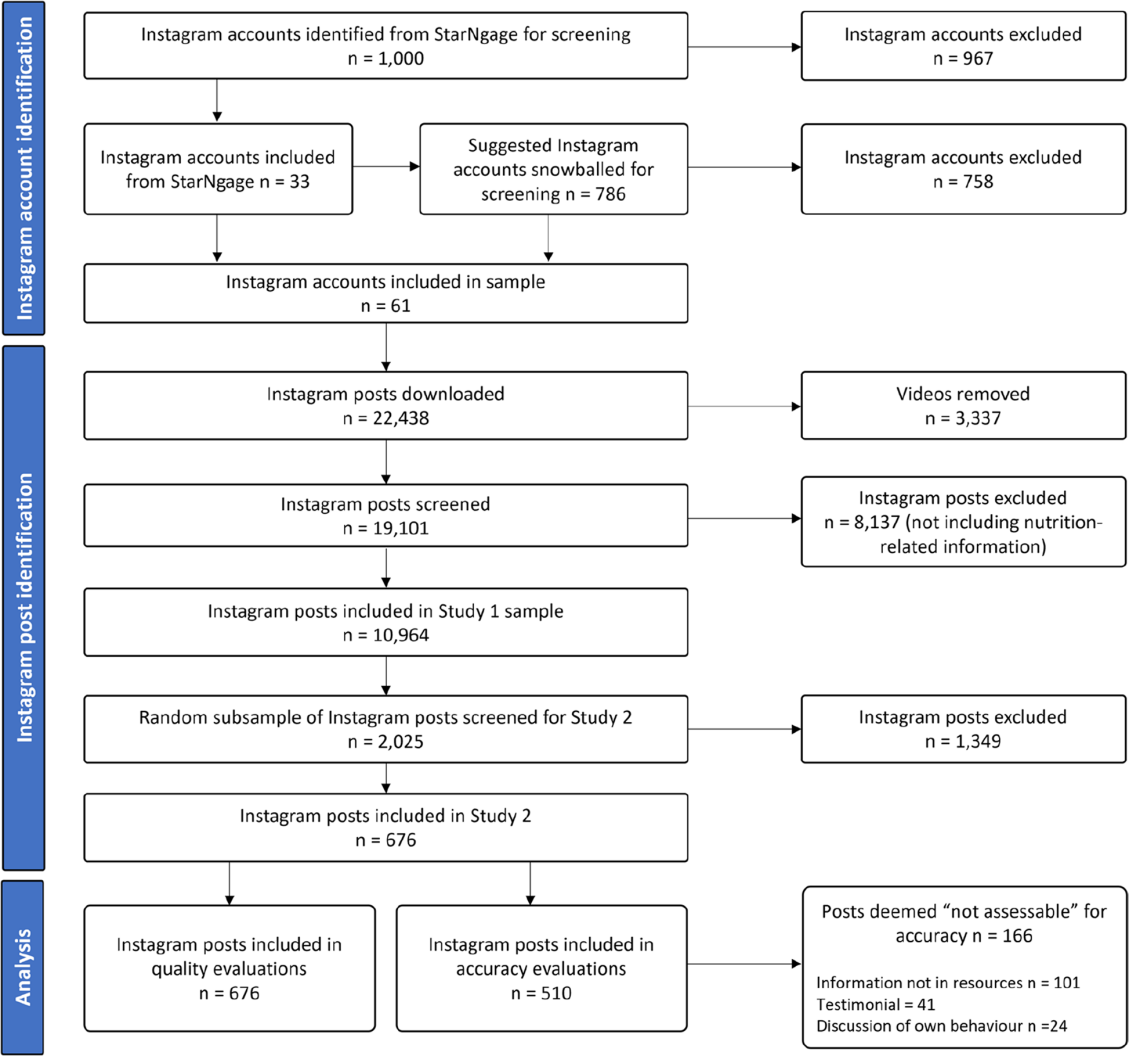
Flow chart of sample selection process

A total of 676 posts from 47 accounts were included in the final sample (Fig. 1, Table 3). Accounts had an average of 314,817 followers. The most common topic discussed in posts was supplements (36.4%) followed by foods/nutrients and health (17.2%), general healthy eating (16.0%), weight loss (13.5%), sports/exercise nutrition (6.7%), other (5.5%) and paediatric nutrition (4.9%).

**Table 3:** Characteristics of Instagram accounts (*n* = 47) and Instagram posts (*n* = 676) included in total sample

Instagram account category (*n*)	Total posts in sample *n* (%)	Followers (mean ± standard deviation)	Post topic *n* (%)						
			**Weight loss**	**Sports/exercise nutrition**	**Supplements**	**Foods/nutrients & health**	**General healthy eating**	**Paediatric nutrition**	**Other**
All (47)	676 (100)	271,789 ± 293,101	91 (13.5)	45 (6.7)	246 (36.4)	116 (17.2)	108 (16.0)	33 (4.9)	37 (5.5)
**By account category**									
Brand (22)	328 (48.5)	230,188 ± 96,768	33 (10.1)	5 (1.5)	208 (63.4)	50 (15.2)	26 (7.9)	0 (0)	7 (2.1)
Fitness/coaching influencer (9)	146 (21.6)	448,946 ± 626,286	46 (31.5)	39 (26.7)	23 (15.8)	12 (8.2)	23 (15.8)	0 (0)	3 (2.1)
Lifestyle influencer (8)	57 (8.4)	285,787 ± 152,421	3 (5.3)	0 (0)	11 (19.3)	18 (31.6)	19 (33.3)	0 (0)	6 (10.5)
Nutritionist/dietitian influencer (8)	145 (21.4)	172,888 ± 88,051	9 (6.2)	1 (0.7)	4 (2.8)	36 (24.8)	41 (28.3)	33 (22.8)	21 (14.5)

### Quality

Overall, 34.8% (*n* = 235) of posts were classified as poor, 59.2% (*n* = 400) mediocre, 6.1% (*n* = 41) good and zero posts were classified as excellent quality. The mean PRHISM score was mediocre (31.8 ± 10.3 out of a possible score of 100) and scores varied across principles (Table 4). Only two principles had a mean score above two (4. Financial disclosure and 13. Images) (Table 5). Posts authored by accounts in the nutritionist/dietitian influencer account category were associated with higher quality scores compared to the reference group (brands) (β, 17.8, CI 13.94–21.65; *P* < 0.001) and all other account categories. Posts that contained information about foods/nutrients and health or general healthy eating were associated with higher quality scores compared to the reference group (weight loss information) and posts containing information about supplements. There was no association between information quality and accounts being verified (β 0.572, *P* = 0.821) or follower count (β -1.18, *P* = 0.643). Lower quality scores were associated with higher engagement (β -0.59, *P* = 0.012) and higher engagement rate (β -0.57, *P* = 0.016), although these differences were small. A complete summary of the results from the mixed effects analysis of quality scores by account type and post topic is provided in Supplementary Table 3 and 4.

**Table 4:** Number and percent of posts categorised according to quality (*n* = 676) and accuracy (*n* = 510) with scores across account categories and post topics

	Engagement* (n = 676)	Quality (as rated by PRHISM)**					**Accuracy**				
		**Posts n**	**Poor n (%)**	**Mediocre n (%)**	**Good n (%)**	**Excellent n (%)**	**Posts n**	**Completely inaccurate n (%)**	**Mostly inaccurate, some accurate n (%)**	**Mostly accurate, some accurate n (%)**	**Completely accurate n (%)**
**All**	1960 ± 3993	676	235 (34.8)	400 (59.2)	41 (6.1)	0 (0)	510	44 (8.6)	73 (14.3)	111 (21.8)	282 (55.3)
**Account category**											
Brand	720 ± 1153	328	155 (47.2)	173 (52.7)	0 (0)	0 (0)	241	29 (12.0)	62 (25.7)	49 (20.3)	101 (41.9)
Fitness/coaching influencer	4368 ± 6910	146	54 (37.0)	92 (63.0)	0 (0)	0 (0)	104	8 (7.7)	10 (9.6)	18 (17.3)	68 (65.4)
Lifestyle influencer	2420 ± 3293	57	25 (43.9)	32 (56.1)	0 (0)	0 (0)	40	4 (10)	5 (12.5)	7 (17.5)	24 (60.0)
Nutritionist/dietitian influencer	2144 ± 3137	145	1 (0.7)	103 (71.0)	41 (28.3)	0 (0)	125	3 (2.4)	9 (7.2)	24 (19.2)	89 (71.2)
**Post topic**											
Weight loss	2652 ± 3496	91	34 (26.4)	57 (62.6)	0 (0)	0 (0)	66	2 (3.0)	4 (6.1)	19 (28.8)	41 (62.1)
Sports/exercise nutrition	6793 ± 9655	45	19 (42.2)	26 (57.8)	0 (0)	0 (0)	26	0 (0)	1 (3.8)	4 (15.4)	21 (80.8)
Supplements	1021 ± 2420	246	129 (52.4)	116 (47.2)	1 (0.4)	0 (0)	175	28 (16)	49 (28.0)	36 (20.5)	62 (35.4)
Foods/nutrients and health	952 ± 1477	116	28 (24.1)	72 (62.1)	16 (13.8)	0 (0)	97	9 (9.3)	6 (6.2)	21 (21.6)	61 (62.9)
General healthy eating	2212 ± 3541	108	17 (15.7)	77 (71.3)	14 (13.0)	0 (0)	90	2 (2.1)	4 (4.1)	14 (15.5)	70 (77.8)
Paediatric nutrition	2293 ± 4222	33	0 (0)	27 (81.8)	6 (18.2)	0 (0)	29	1 (3.5)	7 (24.1)	10 (34.5)	11 (37.9)
Other (e.g., food sustainability, veganism)	2764 ± 3734	37	8 (21.6)	25 (67.6)	4 (10.8)	0 (0)	27	2 (7.4)	2 (7.4)	7 (25.9)	16 (59.3)

*Engagement was calculated as the total number of likes and comments for each post

**PRHISM scoring: poor 0–25, mediocre 26–50, good 51–75, excellent 76–100

**Table 5:** Quality scores for nutrition-related Instagram posts by principle of the PRHISM tool and overall score

Principle	PRHISM Score		
	**Score**	**Minimum**	**Maximum**
1. Authorship	1.0 ± 1.3	0	4
2. Authoritative	1.1 ± 1.7	0	4
3. Action-orientated	1.8 ± 1.1	0	4
4. Financial disclosure	2.1 ± 1.7	0	4
5. Attribution	0.1 ± 0.5	0	4
6. Balance & justifiability	0.2 ± 0.6	0	4
7. Risks & benefits	1.0 ± 0.6	0	4
8. Privacy	0.2 ± 0.6	0	1
9. Complementary information	0.1 ± 0.6	0	4
10. Referrals & support	1.5 ± 0.9	0	4
11. Readability & comprehensibility	1.8 ± 1.2	0	4
12. Accessibility	1.8 ± 0.7	0	4
13. Images	2.7 ± 0.9	1	4
**Overall score**	31.8 ± 10.3	14.6	72.7

Each principle in PRHISM is scored from 0 to 4, where a higher score indicates higher quality. Total PRHSIM scores are from 0–100, where a higher score indicates higher quality.

## Accuracy

From the 676 posts included in the sample, 166 were not assessable and a total of 510 posts were evaluated for accuracy (see Fig. 1). Of the posts that were evaluated for accuracy, 44.7% (*n* = 228) of posts contained inaccuracies, 8.6% (*n* = 44) of posts were completely inaccurate, 14.3% (*n* = 73) mostly inaccurate, 21.8% (111) mostly accurate and 55.3% (*n* = 282) completely accurate. Posts published by fitness influencers had higher odds of receiving a higher score for accuracy compared to posts published by brands (OR 3.09, CI 1.21–7.87, *P* = 0.018) as did posts published by nutritionist/dietitian influencer accounts (OR 4.69, CI 1.81–12.14, *P* = 0.001). In terms of topics, posts containing information about supplements had lower odds of receiving a higher accuracy score compared to posts containing information about weight loss (OR 0.23, CI 0.10–0.51, *P* = 0.00), sports/exercise nutrition, foods/nutrients and health, and general healthy eating. There was no difference in odds of receiving a higher accuracy score for posts authored by a verified or non-verified account (OR 2.07, CI 0.87–4.94, *P* = 0.10), follower count (OR 1.12, CI 0.46–2.69, *P* = 0.803), engagement (OR 1.02, CI 0.84–1.24, *P* = 0.831) or engagement rate (OR 1.0, CI 0.82–1.21, *P* = 0.960). A complete summary of the results from the mixed effects analysis of accuracy scores by account type and post topic is provided in Supplementary Table 3 and 4.

Posts that were completely accurate were associated with higher quality scores compared to posts that were completely inaccurate (β 2.24, CI 0.29–4.19, *P* = 0.024). However, the differences in quality scores between posts containing completely accurate versus completely inaccurate information was small. There was no difference in quality scores observed for posts containing mostly inaccurate or mostly accurate information.

## Discussion

This content analysis study evaluated the quality and accuracy of nutrition-related information posted by popular Australian Instagram accounts. Results indicate that most information posted by Australian accounts is of low to moderate quality and almost half of posts contain inaccuracies. Information about supplements or posted by brand accounts tended to be of lower quality and accuracy compared to other topics and authors of information. Australian nutritionist and dietitian accounts posted higher quality information that was more likely to be accurate.

Overall, the quality and accuracy of nutrition-related Instagram posts included in this study was poor and posts that were of lower quality received higher engagement. These findings are consistent with content analyses of nutrition-related information from YouTube, [48–51] Instagram, [36] WhatsApp, [52] Twitter, [53] Facebook, [54] and blogs, [55] which found large proportions of inaccurate and poor quality information. Furthermore, a study on YouTube videos about nutrition following bariatric surgery found that poor quality and inaccurate videos were the most popular [50]. Conversely, studies about healthy eating information on blogs [56] and information about food safety and eating for coeliac disease on YouTube [57, 58] have found information to be accurate and of fair to high quality. More broadly, studies about health-related information on social media have found that health misinformation is abundant on social media platforms and is often more popular than factual information [31]. A small association between information accuracy and quality was observed, which is consistent with previous research that has seen a very weak correlation [59] or no correlation [60] between the quality and accuracy of nutrition-related information. Findings from this study and the health and nutrition communication literature suggest that Instagram and other social media users are likely to be exposed to suboptimal and misleading nutrition information. Furthermore, over a third of posts in this study contained a combination of accurate and inaccurate information, which may make it difficult for consumers to identify accurate information when engaging with nutrition-related posts by Australian accounts. The generally low PRHISM scores that Australian accounts received suggest that consumers may be presented with information that is difficult to understand and lacks sufficient context or evidence, which may undermine public health nutrition efforts and contribute to confusion and backlash [34, 35].

Information about supplements and information posted by brand accounts was of the lowest quality and accuracy consistent with existing research. A systematic review of the quality and accuracy of online nutrition information found that information published by commercial entities was often of the lowest quality and accuracy, however, this finding was not consistent throughout the included studies and there was variation in which publishers provided the most reliable information [25]. The same review also found that information about supplements was typically inaccurate and of low quality [25]. Similarly, Basch et al. found that YouTube videos about multivitamin supplements were of poor quality, [49] and a small analysis of Instagram posts containing #immunebooster found numerous inaccurate claims about the immune boosting benefits of supplements during the COVID-19 pandemic [61]. Marketing from brands and influencers dominates social media and the marketing of supplements is prevalent on Instagram [4, 7]. The inaccurate and poor-quality information about supplements and posted by Australian brand accounts observed in this study may indicate that exaggerated information may be used as a marketing tactic on Instagram. While consumers should be critical of nutrition information provided by commercial entities or alongside the marketing of supplements and other products, greater regulation is also required to protect consumers from commercial interests that perpetuate misinformation. In 2022, an Australian supplement company with a large social media presence was fined $26,640 AUD by the Therapeutic Goods Administration for unlawful claims about their supplements and cancer and Alzheimer’s prevention [62]. More frequent and severe prosecution for misleading information may help disincentivise commercial entities from making false claims.

In this study posts by Australian nutritionists and dietitians generally received higher quality and accuracy scores than posts by other accounts. This is consistent with results from a recent analysis of nutrition-related Instagram content, where posts categorised as “nutrition and dietetics” received higher quality evaluations compared to other categories such as “fitness” and “motivation” [36]. Furthermore, previous accuracy evaluations of website content authored by registered dietitians versus nutritionists found that dietitians provided more accurate information in two studies based in Canada and the United States, where the title “nutritionist” is not regulated [63, 64] However, contrasting results were observed in an international analysis of tweets by dietitians, which found that 58% of tweets were not evidence based [53].

Although information posted by Australian nutritionists and dietitians was of the highest quality and accuracy in this study, no posts were classified as excellent quality and inaccuracies were detected in over a quarter of their posts. In recent years, the public’s trust in nutrition science has generally eroded [65, 66]. Factors such as scientific uncertainty, conflicts of interest – both real and perceived, and insufficient context and contradictory messaging in nutrition communication have diminished the public’s trust in credible and authoritative voices in nutrition science.[65, 66] It is important for nutrition experts to post high-quality and accurate information to prevent the worsening of mistrust in nutrition science. The quality scores observed may reflect the higher engagement received by posts that were lower in quality and nutrition professionals may be developing content that conforms with what is popular on social media to increase their reach and engagement. Regardless, Australian nutritionists and dietitians should improve the quality of their posts by including references, referring readers to relevant health professionals, and ensuring information is accessible, avoids jargon and is written at an appropriate reading level [30, 67]. In this study it was rare for nutrition professionals to adequately describe their qualifications in their Instagram bios. Providing more information about education and accreditation may improve the quality of nutrition professionals’ health communication and indicate their expertise to consumers [30]. However, increasing transparency regarding qualifications may not result in increased uptake of nutrition advice from credentialed experts, given the public’s diminished trust in the field.

This study had a number of key strengths. Use of the PRHISM tool to evaluate information quality is a strength as it was designed for social media content and measures aspects of information quality that are unique to social media [43]. Typically, studies evaluating the quality of health or nutrition-related social media content have used tools developed for different contexts, which may not be appropriate for social media [25]. All posts were screened by two researchers and a random 10% of posts were evaluated twice for reliability. Furthermore, this study included a large sample of posts collected over a 12-month period, which may improve the generalisability of results. There are also limitations to consider. Firstly, a portion of posts were coded as “not assessable” because the information in the post was not contained in the resources used to review accuracy. As such, the amount of inaccurate information may have been underestimated because only common nutrition myths were contained in the resources and less common inaccuracies were not. Secondly, agreement on reliability measures for quality and accuracy evaluations was moderate. However, protocols were improved based on common disagreements and the remainder of evaluations were done by one author to improve consistency. Thirdly, it is not possible to determine the influence of bots on follower counts or engagement or if the Instagram accounts included in the sample was comprehensive of all prominent accounts that post nutrition-related information. This is because bots are difficult to detect and Instagram restricts access to their application programming interface (API), meaning that much of Instagram’s data cannot be accessed or systematically searched. However, limited access to APIs and the influence of algorithms and bots are common limitations in social media research. Fourthly, the Instagram data used in this study was sourced from Australian Instagram accounts and may therefore not be generalisable to nutrition-related Instagram content published in different geographical locations. Finally, Reels and other video content was not included in this study because it was not feasible to transcribe video content for analysis. Reels have grown in popularity since the data was collected [68] and future research should investigate the quality and accuracy of nutrition-related Instagram Reels.

Findings from this content analysis have implications for policy, practice, and future research. In Australia regulatory bodies have handed down fines for misleading claims, [62] prohibited influencer marketing of therapeutic goods, such as supplements, [69] and put a call out to social media users to report influencers who do not disclose brand partnerships [70]. These are promising steps toward curbing unreliable health and nutrition misinformation. However, regulatory bodies and the public should not bear all responsibility and do not have sufficient resources for comprehensive surveillance and monitoring of social media and the findings from this study highlight the failure of current public health measures to adequately tackle this issue in Australia. Social media companies should do more to regulate content on their platforms, which is strongly recommended by the World Health Organization [71]. For example, social media platforms could verify the qualifications of health professionals and introduce features that enable content creators to easily include references in posts and refer individuals to local health organisations to improve credible communication that aligns with PRHISM and World Health Organization recommendations [30, 71]. Furthermore, the current system that social media, food, wellness and supplement companies operate within, prioritises profit over human and planetary health and is acknowledged within commercial determinants of health framework [72]. A substantial reorientation of this system to prioritise health over profit may be more effective for preventing health misinformation than reprimanding individual influencers or brands for misleading consumers. Nutrition experts also have a role to play and should ensure that the content they publish on social media is accurate and of a high quality. Support for nutritionists, dietitians, and other experts can be provided by professional bodies and institutions to embed media and communications training within tertiary education and continuing professional development. Nutrition communication has been outlined as a priority area in the Australian National Committee for Nutrition decadal plan for the science of nutrition, highlighting the importance of communication to the field of nutrition [73]. Future research should characterise who is exposed to nutrition misinformation, and who engages with, shares and believes misinformation. Additionally, more work is needed to understand how nutrition misinformation may be influencing the dietary choices consumers are making. Further analysis by topic may also yield helpful insights. Considering the importance of infant nutrition, the paediatric topic area could be a significant are to focus future efforts. Finally, research is needed to develop methods of measuring health misinformation’s severity and potential for harm so that potential impacts can be estimated.

## Conclusion

This content analysis found that a large proportion of nutrition-related information posted by influential Australian Instagram accounts is of suboptimal quality and accuracy. Instagram users who follow and engage with nutrition-related Instagram content posted by Australian influencers and brands may be at risk of being misinformed. Information about supplements and content posted by brand accounts was more likely to contain inaccuracies and be of lower quality. Posts by dietitians and nutritionists were higher in quality and more likely to be accurate. The public should be sceptical of the credibility of nutrition-related Instagram content that includes marketing and seek out information provided by nutritionists and dietitians over other entities on Instagram. Although information posted by Australian dietitians and nutritionists was of the highest quality and accuracy, there is scope for improvement and nutrition experts should prioritise providing credible and reliable nutrition communication on social media.

### Supplementary Information

**Additional file 1: Supplementary Table 1.** Strengthening the Reporting of Observational Studies in Epidemiology (STROBE) checklist. **Supplementary Table 2.** Description of information topics and Instagram account categories. **Supplementary Table 3.** Association between quality scores, accuracy scores and Instagram account category. **Supplementary Table 4.** Association between quality scores, accuracy scores and topic of Instagram post

## Data Availability

The data for this study is not publicly available because it was not possible to completely de-identify the content of the Instagram posts and some brands and individuals are identifiable in the dataset. Data can be made available upon reasonable request to the corresponding author.

## Abbreviations

ADG: Australian Dietary Guidelines
NRV: Nutrient Reference Values
PEN: Practice-based Evidence in Nutrition
PRHISM: Principles for Health-related Information on Social Media
STROBE: Strengthening the Reporting of Observational Studies in Epidemiology
