# Supplementary material for: #Fail: the quality and accuracy of nutrition-related information by influential Australian Instagram accounts
Source: Int J Behav Nutr Phys Act. 2024 Feb 14;21:16. doi: 10.1186/s12966-024-01565-y (PMC10865719; doi:10.1186/s12966-024-01565-y)
Supplement: Supplementary file 1 — Additional file 1: Supplementary Table 1. Strengthening the Reporting of Observational Studies in Epidemiology (STROBE) checklist. Supplementary Table 2. Description of information topics and Instagram account categories. Supplementary Table 3. Association between quality scores, accuracy scores and Instagram account category. Supplementary Table 4. Association between quality scores, accuracy scores and topic of Instagram post [file 12966_2024_1565_MOESM1_ESM.docx]

Supplementary Table 1: Strengthening the Reporting of Observational Studies in Epidemiology (STROBE) checklist (1)

|  | **Item no** | **Recommendation** | **Page no** |
| --- | --- | --- | --- |
| **Title and abstract** | 1 | (a) Indicate the study’s design with a commonly used term in the title or the abstract.  (b) Provide in the abstract an informative and balanced summary of what was done and what was found. | 2  2 |
| **Introduction**  Background/rationale  Objectives | 2  3 | Explain the scientific background and rationale for the investigation being reported.  State specific objectives, including any prespecified hypotheses. | 3-5  5 |
| **Methods**  Study design  Setting  Participants  Variables  Data sources/  measurement  Bias  Study size  Quantitative variables  Statistical methods | 4  5  6  7  8  9  10  11  12 | Present key elements of study design early in the paper.  Describe the setting, locations, and relevant dates, including periods of recruitment, exposure, follow-up, and data collection.  (a) Cross-sectional study—Give the eligibility criteria, and the sources and methods of selection of participants  Clearly define all outcomes, exposures, predictors, potential confounders, and effect modifiers. Give diagnostic criteria, if applicable.  For each variable of interest, give sources of data and details of methods of assessment (measurement). Describe comparability of assessment methods if there is more than one group.  Describe any efforts to address potential sources of bias.  Explain how the study size was arrived at.  Explain how quantitative variables were handled in the analyses. If applicable, describe which groupings were chosen and why.  (a) Describe all statistical methods, including those used to control for confounding.  (b) Describe any methods used to examine subgroups and interactions.  (c) Explain how missing data were addressed.  (d) Cross-sectional study—If applicable, describe analytical methods taking account of sampling strategy.  (e) Describe any sensitivity analyses. | 5-7  5-6  5-6  8  5-7  6-8  6, 9  8  8  8  N/A  8  N/A |
| **Results**  Participants  Descriptive data  Outcome data  Main results  Other analyses | 13  14  15  16  17 | (a) Report numbers of individuals at each stage of study—e.g., numbers potentially eligible, examined for eligibility, confirmed eligible, included in the study, completing follow-up, and analysed.  (b) Give reasons for non-participation at each stage.  (c) Consider use of a flow diagram.  (a) Give characteristics of study participants (e.g., demographic, clinical, social) and information on exposures and potential confounders.  (b) Indicate number of participants with missing data for each variable of interest.  Cross-sectional study—Report numbers of outcome events or summary measures.  (a) Give unadjusted estimates and, if applicable, confounder-adjusted estimates and their precision (e.g., 95% confidence interval). Make clear which confounders were adjusted for and why they were included.  (b) Report category boundaries when continuous variables were categorized.  (c) If relevant, consider translating estimates of relative risk into absolute risk for a meaningful time period.  Report other analyses done—e.g., analyses of subgroups and interactions, and sensitivity analyses | 9  9  9  10-11  N/A  11-13  14-15  12  N/A  N/A |
| **Discussion**  Key results  Limitations  Interpretation  Generalisability | 18  19  20  21 | Summarise key results with reference to study objectives.  Discuss limitations of the study, taking into account sources of potential bias or imprecision. Discuss both direction and magnitude of any potential bias.  Give a cautious overall interpretation of results considering objectives, limitations, multiplicity of analyses, results from similar studies, and other relevant evidence.  Discuss the generalisability (external validity) of the study results. | 16  19  20  19 |
| **Other information**  Funding | 22 | Give the source of funding and the role of the funders for the present study and, if applicable, for the original study on which the present article is based. | 21 |

Supplementary Table 2: Description of information topics and Instagram account categories (2)

| **Account category** | **Description** |
| --- | --- |
| Brand | Accounts that were in the name of a brand or company, rather than an individual e.g., the account for a supplement company or online program/app. |
| Fitness/coaching influencer | Accounts that were in the name of a single individual who primarily posted about fitness, sport, exercise, and nutrition. Often accountholders self-identified as personal trainers or coaches. |
| Lifestyle influencer | Accounts that were in the name of a single individual who primarily posted about their own life, their personal health, routine, and products they use. |
| Nutritionist/dietitian influencer | Accounts that were in the name of a single individual who self-identified as a nutritionist or dietitian. The qualifications of the individual were not verified in this study. |
| **Information topic** | **Description** |
| Weight loss | Information about dieting and eating to achieve weight loss. |
| Sports/exercise nutrition | Information about eating or important nutrients to improve sport or exercise performance. |
| Foods/nutrients and health | Information about the relationship between foods or nutrients and health outcomes, excluding information about supplements. |
| Supplements | Information about supplements and their benefits. |
| General healthy eating | General information about eating for health that is not linked to specific health outcomes, e.g., recommending the number of serves of fruit to eat per day. |
| Pediatric nutrition | Information about infant and child feeding, including breastfeeding and introducing solids. |
| Other | Information that covered nutrition topics that did not fit within other categories but did not occur frequently enough to require a separate category, e.g., food sustainability or specific dietary patterns such as veganism or vegetarianism. |

Supplementary Table 3: Association between quality scores, accuracy scores and Instagram account category

| **Quality** | | | | | | | | | | |
| --- | --- | --- | --- | --- | --- | --- | --- | --- | --- | --- |
| **Account category** | **β [95% CI]** | ***P*** | **Marginal mean PRHISM score** | **Pairwise differences** | | | | | | |
|  |  |  |  | **Brand** | | **Fitness influencer** | | **Lifestyle influencer** | **Nutritionist/ dietitian influencer** | |
| Brand | *Ref* | - | 27.2 | - | | 1.10 | | 1.48 | 17.80** | |
| Fitness influencer | 1.10 [-2.6, 4.83] | 0.564 | 28.3 | - | | - | | 0.39 | 16.70** | |
| Lifestyle influencer | 1.48 [-2.45, 5.42] | 0.460 | 28.6 | - | | - | | - | 16.31** | |
| Nutritionist/dietitian influencer | 17.80 [13.94, 21.65] | 0.000 | 45.0 | - | | - | | - | - | |
| **Accuracy** | | | | | | | | | | |
| **Account category** | **OR [95% CI]** | ***P*** | **Pairwise differences** | | | | | | | |
|  |  |  | **Brand** | | **Fitness influencer** | | **Lifestyle influencer** | | | **Nutritionist/ dietitian influencer** |
| Brand | *Ref* | - | - | | 1.13* | | 0.83 | | | 1.55* |
| Fitness influencer | 3.09 [1.21, 7.87] | 0.018 | - | | - | | -0.30 | | | 0.42 |
| Lifestyle influencer | 2.29 [0.78, 6.71] | 0.129 | - | | - | | - | | | 0.71 |
| Nutritionist/dietitian influencer | 4.69 [1.81, 12.14] | 0.001 | - | | - | | - | | | - |

**P*<0.05, ***P*<0.001, PRHISM scores range from 0-100 where a higher score indicates higher quality

Supplementary Table 4: Association between quality scores, accuracy scores and topic of Instagram post

| **Quality** | | | | | | | | | | | | | | | |
| --- | --- | --- | --- | --- | --- | --- | --- | --- | --- | --- | --- | --- | --- | --- | --- |
| **Post topic** | **β [95% CI]** | ***P*** | **Marginal mean PRHISM score** | **Pairwise differences** | | | | | | | | | | | |
|  |  |  |  | **Weight loss** | **Sport/**  **exercise nutrition** | | **Supplements** | | **Foods/**  **nutrients & health** | | **General healthy eating** | | **Paediatric nutrition** | | **Other** |
| Weight loss | *Ref* | - | 29.78 | - | 0.59 | | -0.58 | | 2.58* | | 2.20* | | 2.53 | | 0.75 |
| Sports/exercise nutrition | 0.579 [-1.57, 2.73] | 0.597 | 30.35 | - | - | | -1.15 | | 2.00 | | 1.62 | | 1.95 | | 0.17 |
| Supplements | -0.576 [-2.66, 1.50] | 0.588 | 29.20 | - | - | | - | | 3.16** | | 2.78* | | 3.10 | | 1.33 |
| Foods/nutrients & health | 2.58 [0.68, 4.52] | 0.009 | 32.36 | - | - | | - | | - | | -0.38 | | -0.05 | | -1.83 |
| General healthy eating | 2.20 [0.30, 4.10] | 0.023 | 31.98 | - | - | | - | | - | | - | | 0.33 | | -1.83 |
| Paediatric nutrition | 2.53 [-2.71, 7.77] | 0.344 | 32.30 | - | - | | - | | - | | - | | - | | 1.78 |
| Other | 0.75 [-1.70, 3.20] | 0.548 | 30.53 | - | - | | - | | - | | - | | - | | - |
| **Accuracy** | | | | | | | | | | | | | | | |
| **Post topic** | **OR [95% CI]** | ***P*** | **Pairwise differences** | | | | | | | | | | | | |
|  |  |  | **Weight loss** | **Sport/**  **exercise nutrition** | | **Supplements** | | **Foods/**  **nutrients & health** | | **General healthy eating** | | **Paediatric nutrition** | | **Other** | |
| Weight loss | *Ref* | - | - | 0.70 | | -1.49** | | -0.28 | | 0.36 | | -1.32 | | -0.53 | |
| Sports/exercise nutrition | 2.01 [0.6, 6.60] | 0.249 | - | - | | -2.19** | | -0.98 | | -0.34 | | -2.02* | | -1.23* | |
| Supplements | 0.23 [0.10, 0.51] | 0.000 | - | - | | - | | 1.21** | | 1.85** | | 0.17 | | 0.95* | |
| Foods/nutrients & health | 0.76 [0.33, 1.70] | 0.498 | - | - | | - | | - | | 0.64 | | -1.04 | | -0.25 | |
| General healthy eating | 1.43 [0.61, 3.38] | 0.415 | - | - | | - | | - | | - | | -1.68* | | -0.89* | |
| Paediatric nutrition | 0.27 [0.05, 1.32] | 0.105 | - | - | | - | | - | | - | | - | | -0.78 | |
| Other | 0.59 [0.21, 1.64] | 0.310 | - | - | | - | | - | | - | | - | | - | |

**P*<0.05, ***P*<0.001, PRHISM scores range from 0-100 where a higher score indicates higher quality

**References**

1. Von Elm E, Altman DG, Egger M, Pocock SJ, Gøtzsche PC, Vandenbroucke JP. The Strengthening the Reporting of Observational Studies in Epidemiology (STROBE) statement: guidelines for reporting observational studies. Journal of clinical epidemiology. 2008;61(4):344-9.

2. Denniss E, Lindberg R, McNaughton SA. Nutrition-Related Information on Instagram: A Content Analysis of Posts by Popular Australian Accounts. Nutrients. 2023;15(10):2332.
